# Hyperpolarization‐activated cyclic nucleotide‐gated channels working as pacemaker channels in colonic interstitial cells of Cajal

**DOI:** 10.1111/jcmm.17087

**Published:** 2021-11-29

**Authors:** Seok Choi, Hyunhyo Seo, Kyungmin Lee, Dong Hoon Shin, Mei Jin Wu, Wenhao Wu, Xingyou Huang, Jingwei Zhang, Chansik Hong, Jae Yeoul Jun

**Affiliations:** ^1^ Department of Physiology College of Medicine Chosun University Gwangju Korea; ^2^ Department of Anatomy Brain Science & Engineering Institute School of Medicine Kyungpook National University Daegu Korea

**Keywords:** hyperpolarization‐activated cyclic nucleotide‐gated channels, interstitial cells of Cajal, pacemaker potential

## Abstract

Hyperpolarization‐activated cyclic nucleotide‐gated (HCN) channels function as pacemaker channels in spontaneously active cells. We studied the existence of HCN channels and their functional roles in the interstitial cells of Cajal (ICC) from the mouse colon using electrophysiological, immunohistochemical and molecular techniques. HCN1 and HCN3 channels were detected in anoctamin‐1 (Ca^2+^‐activated Cl^−^ channel; ANO1)‐positive cells within the muscular and myenteric layers in colonic tissues. The mRNA transcripts of HCN1 and HCN3 channels were expressed in ANO1‐positive ICC. In the deletion of HCN1 and HCN3 channels in colonic ICC, the pacemaking potential frequency was reduced. Basal cellular adenylate cyclase activity was decreased by adenylate cyclase inhibitor in colonic ICC, whereas cAMP‐specific phosphodiesterase inhibitors increased it. 8‐Bromo‐cyclic AMP and rolipram increased spontaneous intracellular Ca^2+^ oscillations. In addition, Ca^2+^‐dependent adenylate cyclase 1 (AC1) mRNA was detected in colonic ICC. Sulprostone, a PGE_2_‐EP_3_ agonist, increased the pacemaking potential frequency, maximum rate of rise of resting membrane in pacemaker potentials and basal cellular adenylate cyclase activity in colonic ICC. These results indicate that HCN channels exist in colonic ICC and participate in generating pacemaking potentials. Thus, HCN channels may be therapeutic targets in disturbed colonic motility disorders.

## 1 INTRODUCTION

The diverse membrane ion channels generate electrical activity and determine the firing rate of spontaneously active cells such as cardiac, neuronal and endocrine cells. Hyperpolarization‐activated cyclic nucleotide (HCN) channels in these cells function as pacemaking channels that cause diastolic depolarization to initiate rhythmic activity. Interstitial cells of Cajal (ICC) are also active cells in the gastrointestinal (GI) tract that spontaneously generate electrical activity.^1^ ICC form gap junctions with each other or smooth muscle cells.^2^ Thus, spontaneous electrical activity, known as a pacemaker activity, of ICC is directly transmitted to smooth muscles and causes slow waves that determine smooth muscle contractility.^3^ In ICC, a variety of ion channels have been reported, including transient receptor potential (TRP) channels,^4^ Ca^2+^‐activated Cl^−^ channels,^5^ voltage‐dependent Ca^2+^ channels,^6^, ^7^ voltage‐dependent K^+^ channels,^8^ ERG channels,^9^ Na^+^ channels^10^ and ATP‐sensitive K^+^ channels.^11^, ^12^ Among these channels, TRP channels and Ca^2+^‐activated Cl^−^ channels (Anoctamin‐1; ANO1) have been suggested as pacemaker channels in ICC.^13^, ^14^ Particularly, ANO1 is a strong candidate as a pacemaker channel because of the expression of ANO1, failing of slow waves in ANO1 knockout mouse, blocking of slow waves by Ca^2+^‐activated Cl^−^ channel blockers in intact mouse and human intestine and single‐channel recording of Ca^2+^‐activated Cl^−^ channels in ICC.^14^, ^15^

Hyperpolarization‐activated cyclic nucleotide (HCN) channels are pacemaking channels that cause diastolic depolarization to initiate rhythmic activity in cardiac and neuronal cells.^16^, ^17^, ^18^ Previously, we reported that HCN channels might be involved in the generation of pacemaker activity in colonic ICC but not intestinal ICC of mice.^19^ HCN channels are gated by intracellular cAMP directly.^20^ In colonic ICC, adenylate cyclase inhibitors reduced pacemaking potential frequency while cAMP‐specific phosphodiesterase inhibitors enhanced pacemaking potential frequency. In addition, HCN channel blockers inhibited the pacemaker activity, and mRNA transcripts of HCN channels were expressed in colonic ICC.^19^ Sulprostone, a PGE_2_‐EP_3_ agonist, also enhanced the pacemaking potential frequency of colonic ICC, and the enhanced effects were blocked by HCN channel blockers.^21^ Thus, we suggest that HCN channels are candidate pacemaker channels modulated by endogenous substances in colonic ICC. However, our previous results were mainly obtained using pharmacological studies. Thus, to confirm the existence and functional role of HCN channels in colonic ICC, we further performed electrophysiology, immunohistochemistry and molecular studies.

## 2 MATERIALS AND METHODS

### 2.1 Preparation of cells

Experimental and animal care protocols used in the study were all in accordance with the guiding principles approved by the Ethics Committee of Chosun University and the National Institutes of Health Guide, South Korea, for the Care and Use of Laboratory Animals. Mice had free access to water and were fed a standard mouse diet until the day of the experiment. Balb/C mice (5–8 days old) of either sex were anaesthetized with ether and euthanized by cervical dislocation. The small intestines from 1 cm below the pyloric ring to the cecum were removed and colon from below the cecum to the rectum was removed, and the middle portion of the colon was used. The small intestine and colon were opened along the mesenteric border. The luminal contents were washed away with Krebs–Ringer bicarbonate solution. Tissues were pinned to the base of a Sylgard dish, and the mucosa was removed by dissection. Small strips of small intestinal and colonic muscle were equilibrated in Ca^2+^‐free Hank's solution containing: KCl 5.36 mM, NaCl 125 mM, NaOH 0.34 mM, Na_2_HCO_3_ 0.44 mM, glucose 10 mM, sucrose 2.9 mM and HEPES 11 mM for 30 min. The cells were dispersed with an enzyme solution containing 1.3 mg/ml collagenase (Worthington Biochemical Co), 2 mg/ml bovine serum albumin (Sigma), 2 mg/ml trypsin inhibitor (Sigma) and 0.27 mg/ml ATP. Cells were plated onto sterile glass coverslips coated with murine collagen (2.5 μg/ml Falcon/BD) in 35‐mm culture dishes. The cells were then cultured in smooth muscle growth medium (SMGM; Clonetics Corp.) supplemented with 2% antibiotics/antimycotics (Gibco) and 5 ng/ml urine stem cell factor (SCF, Sigma) at 37°C and 5% CO_2_.

### 2.2 Patch‐clamp recording

The current clamp mode of patch clamp was applied to record pacemaking potentials in colonic ICC‐like cells that showed a network‐like structure in cultures after 2–3 days. The cell culture dishes were mounted on a model TE‐2000s inverted microscope (Nikon). The bath solution used was 3 ml/min, and all experiments were performed at 30°C. Pacemaker potentials were amplified using Axopatch 200 B (Axon Instruments). Data were filtered at 5 kHz and displayed on a computer monitor. Results were later analysed using pClamp and GraphPad Prism version 5.0 (GraphPad Software Inc.).

### 2.3 **Collection of** ICC **and reverse transcription chain reaction (RT‐PCR)**

Total cultured cells or picked cells (below 10 cells) with typical ICC morphology (i.e., triangular or spindle‐shaped with several branches) were sucked into a recording pipette under negative pressure. After this, the samples were expelled into phosphate‐buffered saline by applying positive pressure. After picking, the cells were centrifuged at 9800 *g* at 4°C for 8 min before lysis. Total RNA was isolated using TRIzol reagent (Invitrogen) according to the manufacturer's instructions. Reverse transcription was performed using the PrimeScript™ 1st Strand cDNA Synthesis Kit (6110A, Takara). The resultant cDNA was purified using the Maxime PCR PreMix i‐StarTaq (iNtRON Biotechnology Inc.) and amplified. The following three‐step process was executed for 40 cycles: denaturation at 94°C for 30 s, annealing at 60°C for 30 s and extension at 72°C for 30 s. The PCR products were electrophoresed on a 2% agarose gel and visualized using ethidium bromide staining. All primers used for RT‐PCR are shown in Table 1.

**TABLE 1:** Nucleotide sequences of the primers used for RT‐PCR

Gene	Sequences	Accession No	Size (bp)
Ano‐1	(F) AGG CCA AGT ACA GCA TGG GTA TCA	NM_178642	213
	(R) AGT ACA GGC CAA CCT TCT CAC CAA		
Myosin	(F) GAGAAAGGAAACACCAAGGTCAAGC	NM_010860	264
	(R) AACAAATGAAGCCTCGTTTCCTCTC		
PGP 9.5	(F) GCCAACAACCAAGACAAGCTGGAA	AF172334	213
	(R) GCCGTCCACGTTGTTGAACAGAAT		
HCN1	(F) GAGCACTTCGTATCGTGAGG	NM_010408	206
	(R) GGGAACCAGGAACTGAAGAC		
HCN2	(F) GGTCTCGGACACTTTCTTCC	NM_008226	259
	(R) CAGCAGACTGAGGATCTTGG		
HCN3	(F) CCTCATAGTTCTGCCTGTGG	NM_008227	280
	(R) GACCTCAGCATCTAGTCGTG		
HCN4	(F) GGAGAGATCTGCTTGCTGAC	NM_001081192	235
	(R) GGTAGTTGAAGACGCCTGAG		
AC1	(F) GTTCCTGAGGCGTGGTATTT	NM_009622.2	272
	(R) AGCCATTTGTCCCAAGGATAG		
AC3	(F) CAGGTCTCCCTTCCTCATTAGA	NM_001159536.1	211
	(R) CGAAGGTCAGCCGCATAAA		
AC8	(F) CCATCTACGCAGGTCTCTTTC	NM_001331075.1	213
	(R) GTTCCCTCAGTTCCTTCATCTC		

### 2.4 Immunohistochemistry

Adult C57BL/6 mice were transcardially perfused using 100 mM PBS followed by 4% paraformaldehyde in PBS. The colons were removed and post‐fixed in 4% paraformaldehyde in PBS for one day and then dehydrated in 30% sucrose solution for 48 h. Dehydrated colons were embedded with optimal cutting temperature compound (OCT compound) prior to frozen sectioning and then were sectioned using cryo‐microtome (Leica) at 12 μM thickness. Colon tissue sections were rinsed in phosphate‐buffered saline (PBS). Tissue sections were also permeabilized with 0.1% Triton X‐100 diluted in PBS containing 5% normal horse serum for 2 h at room temperature. Afterwards, the sections were incubated for 24 h at 4°C in the primary antibody (1:50 dilution for each HCN subunit; Alomone Labs, and ANO1; Santa Cruz Biotechnology) diluted in PBS containing 5% normal horse serum. After rinsing them in PBS three times at 10 min each, the sections were incubated with an Alexa Fluor 594‐conjugated chicken anti‐rabbit IgG (Molecular Probes) diluted 1:250 in PBS containing 5% normal horse serum for 2 h at room temperature. Finally, the sections were rinsed in PBS three times at 10 min each and coverslipped with Vectashield (Vector Laboratories). For double immunofluorescence experiments, each of the HCN subunit antibodies was combined with an anti‐ANO1 (1:50; Santa Cruz Biotechnology). After incubation with the primary antibody at 4℃ overnight, the sections were incubated with Alexa Fluor 488‐conjugated chicken anti‐goat IgG diluted 1:200 in PBS containing 5% normal horse serum for 2 h at room temperature. Images were obtained using a Zeiss LSM5 confocal microscope using 5× and 20× objective lenses. For each antigen, all colon sections were processed identically, and images were obtained using the same microscope parameters (pinhole;159). The relative intensity of immunopositive signals for HCN1, HCN2, HCN3 and HCN4 subunits in the colon was measured using ImageJ software.^22^ HCN immunolabelling of ANO1‐positive cells was also assessed using confocal microscope imaging.

### 2.5 Measurement of adenylate cyclase activity

Adenylate cyclase activity was measured in cultured ICC. ICC were rinsed twice with ice‐cold PBS and collected in PBS. ICC were homogenized with five strokes of a homogenizer. The homogenate was then centrifuged at 1000 *g* for 5 min at 4°C. The supernatant was then transferred into a centrifuge tube and centrifuged at 5000 *g* for 10 min. The pellet was suspended in buffer (30 mM Na‐HEPES, 5 mM MgCl_2_ and 2 mM DTT, pH 7.5) to attain a total protein concentration of approximately 1 mg/ml before being added into tubes containing the drug and adenylate cyclase assay buffer (30 mM Na‐HEPES, 100 mM NaCl, 1 mM EGTA, 10 mM MgCl_2_, 1 mM isobutylmethylxanthine, 1 mM ATP, 10 mM phosphocreatine, 5 μM GTP, 60 U/ml creatine phosphokinase and 0.1% bovine serum albumin, pH 7.5). Adenylate cyclase activity assays were performed using a mouse adenylate cyclase type 6 ELISA kit (MyBiosource Company).

### 2.6 Transient transfection with small interfering RNAs (siRNAs)

In adherence to the manufacturer's instructions for the use of the transfection reagent, ICCs were plated with 60%–70% confluency and transfected into groups. Before transfection, ICCs were starved in FBS‐free SMBM media for 1–2 h. Transfection compounds were prepared for each dish, according to the instructions for Lipofectamine RNAiMAX (Thermo Fisher Scientific); briefly, siRNA (40 nM) was formulated using the Lipofectamine reagent. The recording was performed 24–36 h after transfection. The sequences of HCN1, HCN3, and negative control small interfering RNA (siRNA) are shown in Table 2.

**TABLE 2:** The sequences of HCN1, HCN3 and negative control siRNA

Gene	Sequences
siHCN1‐mus‐536	(F) GCAACUCCGUGUGCUUCAATT
	(R) UUGAAGCACACGGAGUUGCTT
siHCN1‐mus‐1010	(F) GCUGGUUUGUGGUGGACUUTT
	(R) AAGUCCACCACAAACCAGCTT
siHCN1‐mus‐1193	(F) GGGAAGAGAUAUUCCACAUTT
	(R) AUGUGGAAUAUCUCUUCCCT
siHCN3‐mus‐634	(F) CCAUCCCUGUGGAUUAUAUTT
	(R) AUAUAAUCCACAGGGAUGGTT
siHCN3‐mus‐718	(F) GCAUCGUUAGAUUCACCAATT
	(R) UUGGUGAAUCUAACGAUGCTT
siHCN3‐mus‐1008	(F) GCCAUGAGUCACAUGCUAUTT
	(R) AUAGCAUGUGACUCAUGGCTT

### 2.7 Measurement of intracellular Ca^2+^ concentration

Changes in intracellular Ca^2+^ ([Ca^2+^]_i_) concentrations were monitored using fluo‐4/AM pre‐dissolved in DMSO and stored at –20°C. ICC cultured on coverslips were rinsed twice with the bath solution mentioned above and incubated in a bath solution containing 5 μM fluo‐4/AM under 5% CO_2_ for 5 min at 37°C. Following two more rinses, the cells were mounted on a perfusion chamber and scanned under a confocal microscope every 0.4 seconds (200×; Fluoviews 300, Olympus). Excitation and emission wavelengths of 488 nm and 515 nm, respectively, were used for fluorescence imaging. Variations in [Ca^2+^]_i_ fluorescence emission intensity were expressed as F1/F0 with F0 used as the intensity for the first imaging. The temperature of the perfusion chamber containing the cultured ICC was maintained at 30°C.

### 2.8 Reagents

Cells were bathed in a buffer composed of 5 mmol/L KCl, 135 mmol/L NaCl, 2 mmol/L CaCl_2_, 10 mmol/L glucose, 1.2 mmol/L MgCl_2_ and 10 mmol/L HEPES with the pH adjusted to 7.2 using Tris. The pipette solution was composed of 140 mmol/L KCl, 5 mmol/L MgCl_2_, 2.7 mmol/L K_2_ATP, 0.1 mmol/L Na_2_GTP, 2.5 mmol/L creatine phosphate disodium, 5 mmol/L HEPES and 0.1 mM EGTA with the pH adjusted to 7.2 with Tris. The drugs used were SQ‐22,536, rolipram, 8‐bromo‐cAMP and sulprostone. Solvent for All drugs were DMSO and all drugs were purchased from Sigma‐Aldrich.

### 2.9 Statistical analysis

Data are expressed as mean ± SEM. Student's *t*‐test for paired data was used to assess the significance of any differences observed. Statistical significance was set at *p *< 0.05. The *n*‐values reported in the text refer to the number of cells used in the patch‐clamp experiments.

## 3 RESULTS

### 3.1 Expression of HCN channels in interstitial cells of Cajal

**FIGURE 1:**
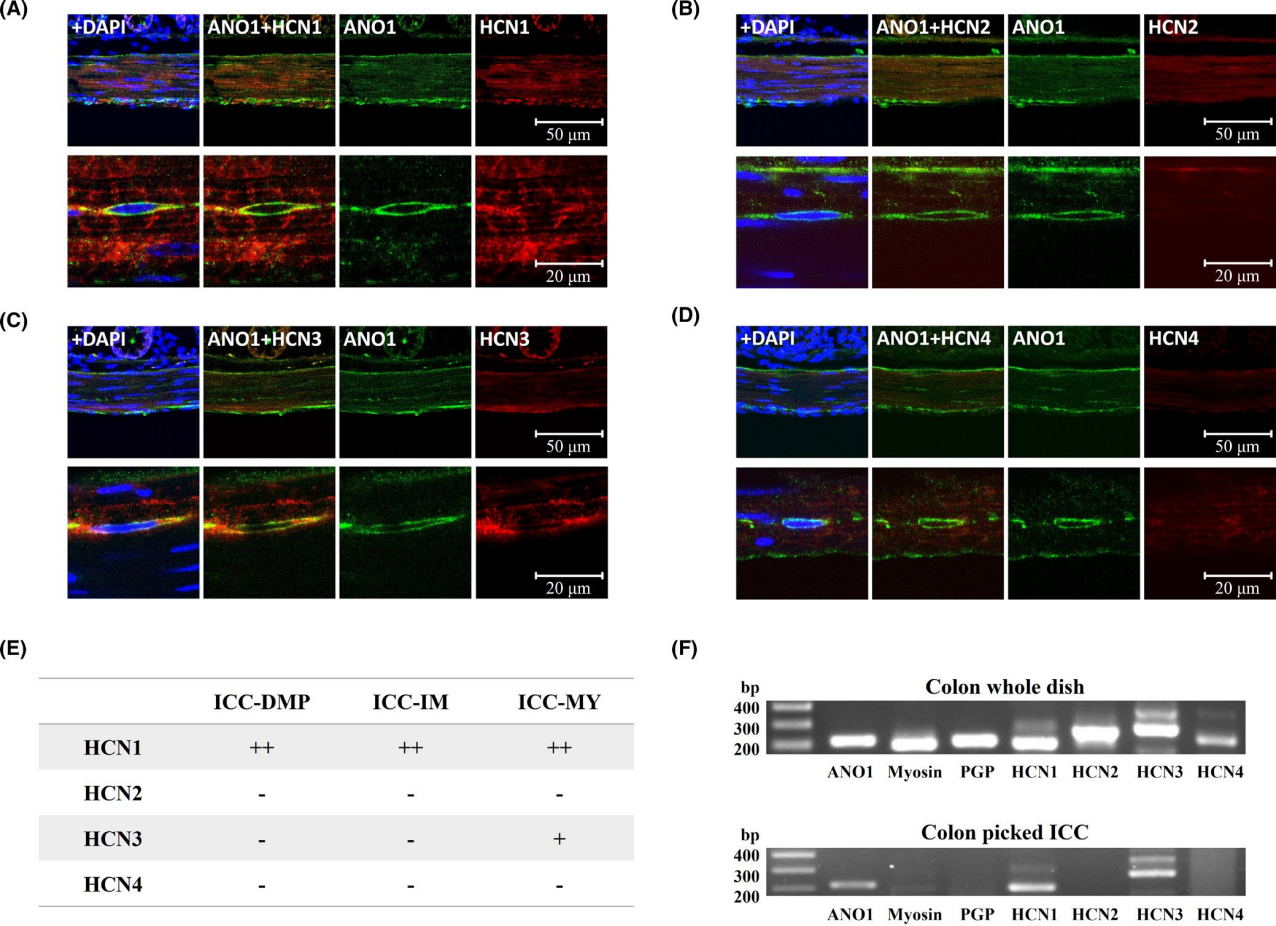
Expression of HCN subunits and ANO1 in mouse colon. (A–D) Upper panels, low magnification images showing whole layers of muscular and myenteric regions. Scale bar, 50 μm Lower panels, high‐magnified images of HCN and ANO1 immunoreactivities in the myenteric layer. Scale bar, 20 μm (A) The expressions of HCN1 are strong in muscular and myenteric regions (upper panels) and HCN1 signals are colocalized in ANO1‐positive ICC of the myenteric layer (lower panels). (B) HCN2 is observed with low expression levels at lower parts of longitudinal layer (upper panels) and HCN2 signal is not found in the myenteric layer (lower panels). (C) HCN3 and ANO1 are found only at myenteric layer (upper and lower panels). (D) Neither HCN4 nor ANO1 is exhibited in the muscular and myenteric layers (upper and lower panels). (E) Summary of expression of HCN subunits in mouse colon. (F) RT‐PCR detection and expression of HCN channel proteins in cultured ICC of mouse colon. Four HCN channel subtypes (HCN1‐HCN4) were amplified in whole mounted, cultured cells from mouse colon. However, only HCN1 and HCN3 channel subtypes were amplified in ANO1‐positive cultured colonic ICC by picking. PGP: PGP 9.5

The expression of HCN channels was investigated using immunohistochemistry studies. Double staining with anti‐ANO1 and HCN antibodies showed HCN1 immunoreactivity in ANO1‐positive ICC of muscular and myenteric layers from mouse colon (Figure 1A, upper panels; Figure S1A, white arrowheads). Lower panels in Figure 1A represent the HCN1 signals in the ANO1‐immunopositive cells of myenteric layers (Figure 1A, lower panels). However, HCN3 immunoreactivity was only seen in the myenteric region in ANO1‐positive ICC (Figure 1C, upper and lower panels; Figure S1C, white arrowhead). Only a low level of HCN2 immunoreactivity was expressed in the longitudinal layer (Figure 1B; Figure S1B), and HCN4 was expressed in neither the myenteric nor muscle layers (Figure 1D; Figure S1D). The summarized distribution of the HCN channels is shown in Figure 1E. To support this, RT‐PCR was performed to detect the expression of HCN channels at the cellular level. RT‐PCR analysis revealed the mRNA transcripts for all four HCN channel subtypes in whole mounted, cultured colonic cells. However, the mRNA transcripts for only HCN1 and HCN3 channels were detected in ANO1‐positive ICC by picking (Figure 1F). These results suggest that HCN channels exist in colonic ICC.

### 3.2 **HCN1 and HCN3 channels mediate the generation of pacemaking activity in cultured colonic** ICC

**FIGURE 2:**
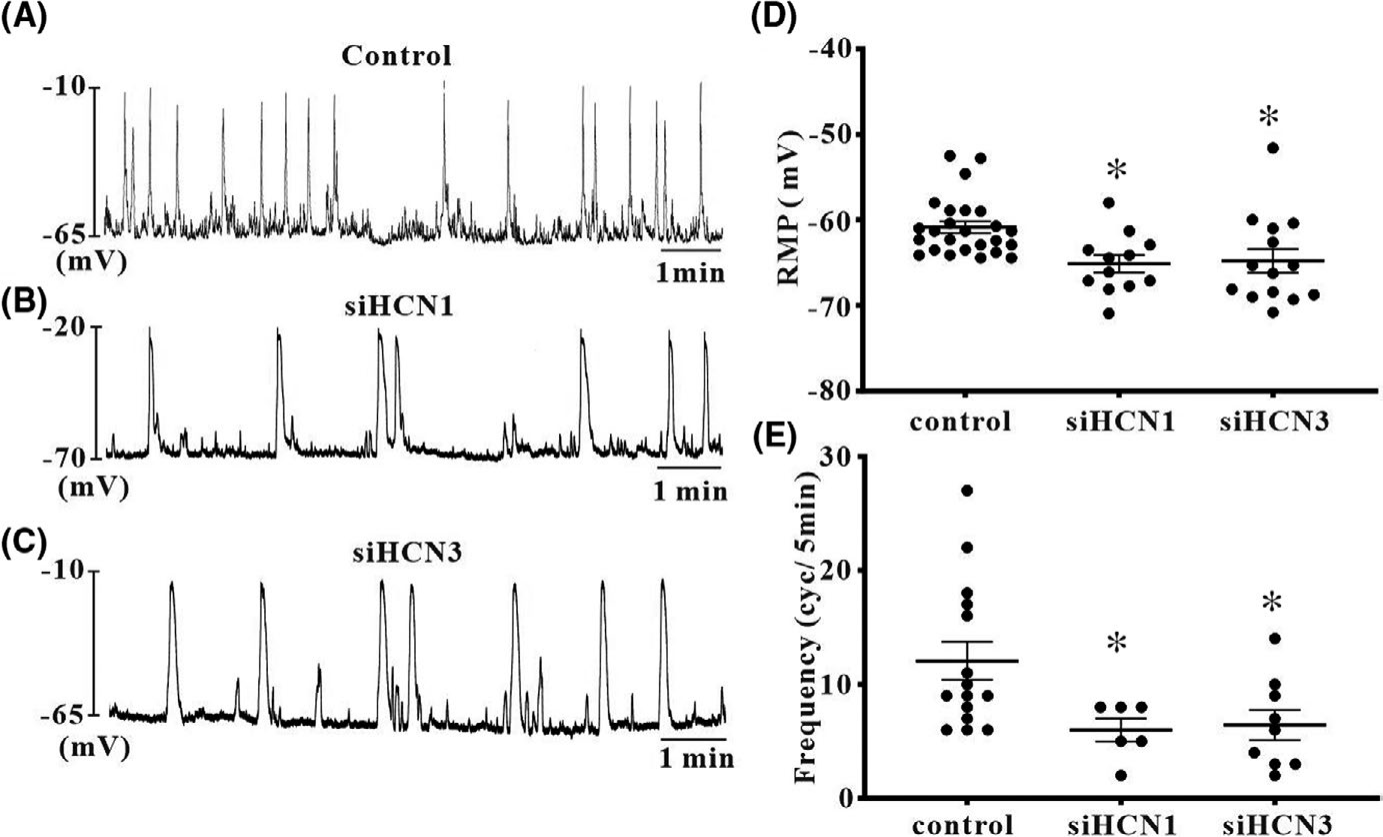
Effects of deletion of HCN1 or HCN3 subunits by siRNA in cultured colonic ICC. (A) Control ICC that infected lipofectamine only showed the typical spontaneous pacemaker potentials. (B and C) ICC that deleted either the HCN1 or HCN3 subunit showed more negative resting membrane potentials and lower pacemaking potential frequency compared with the control. (D and E) Summarized effects of the deletion of HCN1 and HCN3 on membrane potential and pacemaking potential frequency in colonic ICC. The data represent the mean ± SEM. Asterisks indicate values that are significantly different compared with the control values (*p* < 0.05)

**FIGURE 3:**
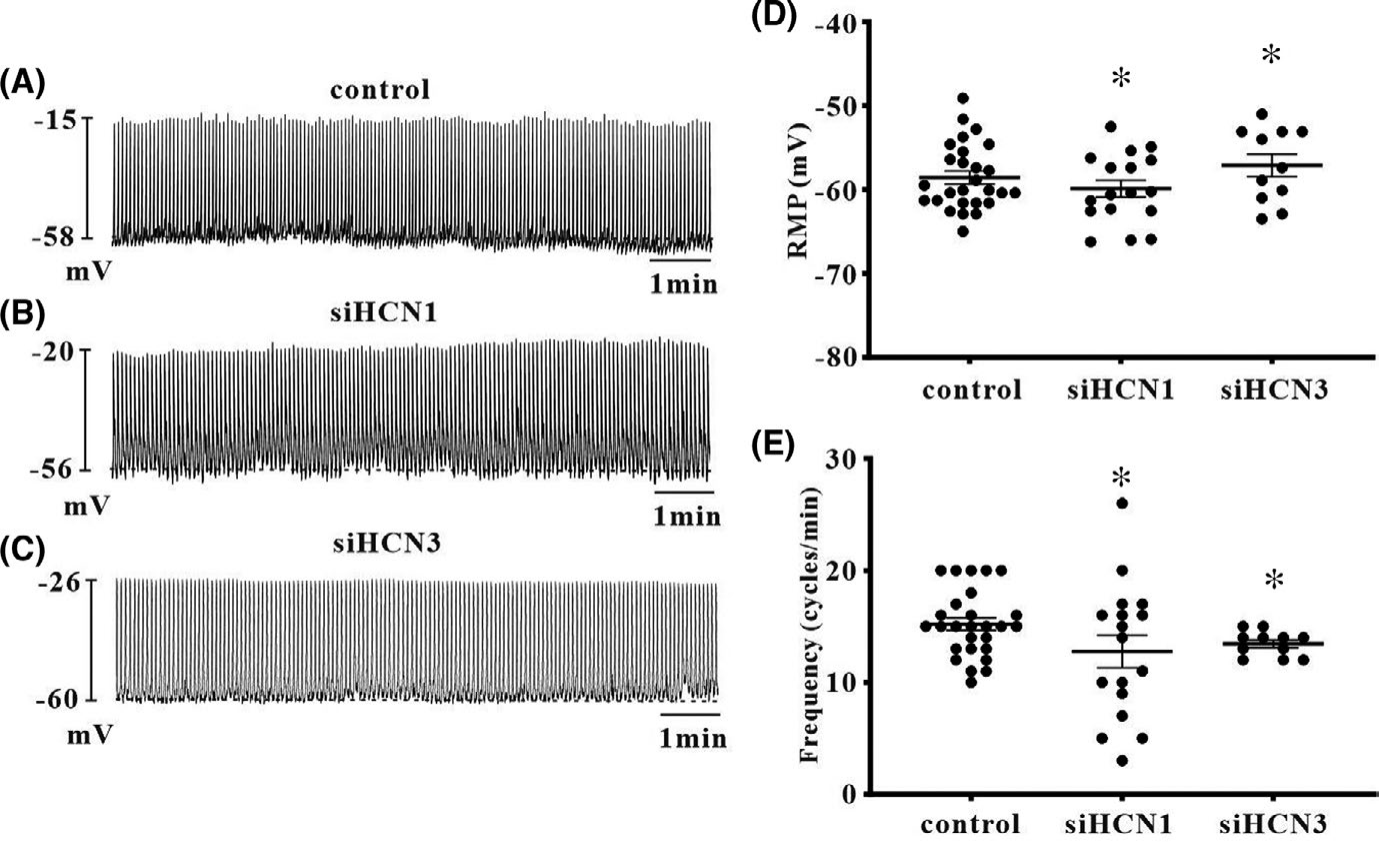
Effects of deletion of HCN1 or HCN3 subunits by siRNA in cultured small intestinal ICCs. (A) The control ICC that infected Lipofectamine only showed the typical periodic spontaneous pacemaker potentials. (B and C) The ICC that deleted HCN1 or HCN3 subunit showed also the periodic spontaneous pacemaker potentials compare to the original behaviour. The deletion of HCN1 or HCN3 has no effects on the generation of pacemaking potential in small intestinal ICC. (D and E) Summarized effects of deletion of HCN1 and HCN3 on the membrane potential and pacemaker potential frequency in small intestinal ICC. The data represent the mean ± SEM

To test whether HCN1 and HCN3 channels function as pacemaker channels, we transfected siRNA vectors of HCN1 and HCN3 channels into cultured ICC after 1 day in culture. In the control group, ICC that transfected Lipofectamine only showed 11.9 ± 2.46 cycles/5 min of pacemaking potential frequency and −61.4 ± 0.68 mV of resting membrane potential (Figure 2A, D, E, *n* = 16). HCN1 or HCN3 siRNA‐transfected ICC showed a reduction in pacemaking potential frequency with slight hyperpolarization (control: siHCN1: siHCN3 = −60.85 ± 0.68: 65.1 ± 1.0: 64.76 ± 1.39 mV) of the resting membrane potential (Figure 2B, C). The values of the resting membrane potential and frequency for each are shown in Figure 2D, E (*n* = 7). However, there were no differences seen in resting membrane potential and frequency between the HCN1 or HCN3 siRNA‐transfected group and control group in small intestinal ICC (Figure 3A–E, *n* = 12–27). These results suggest that HCN channels are involved in generating pacemaking potentials in colonic ICC.

### 3.3 Basal cAMP involved in generating pacemaker potentials in colonic ICC

**FIGURE 4:**
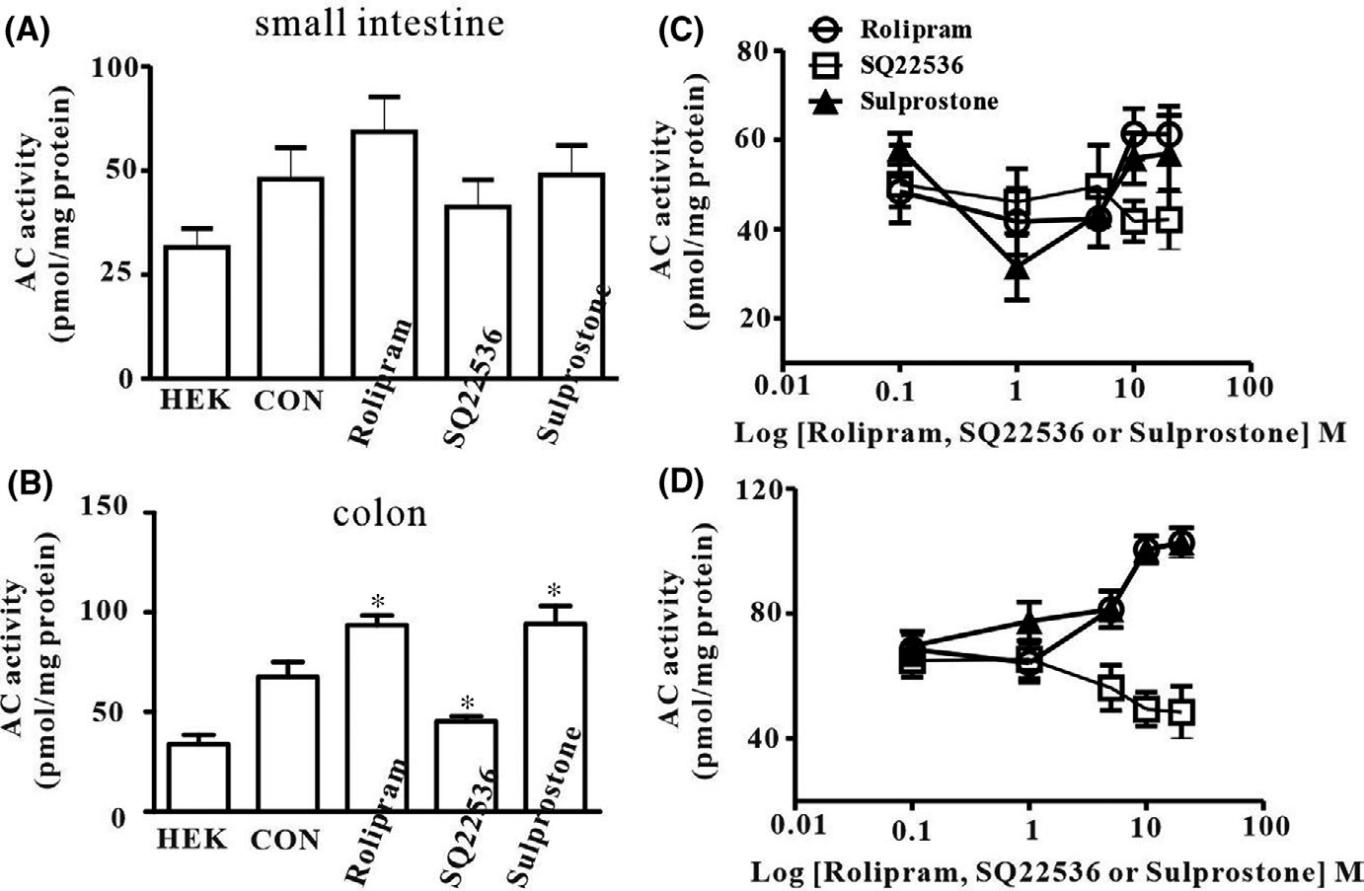
Effects of phosphodiesterase inhibitor, AC inhibitor and sulprostone on basal adenylate cyclase (AC) activity in ICCs. Basal AC activity was measured in HEK cells, small intestinal ICC and colonic ICC. (A) Rolipram (10 μM), a cAMP‐specific phosphodiesterase inhibitor, SQ22536 (100 μM), an AC inhibitor, and sulprostone, a PGE2‐EP3 agonist, did not change basal AC activity in small intestinal ICC. (B) In colonic ICC, rolipram and sulprostone increased basal AC activity while SQ22536 decreased basal AC activity when compared with the control. The data represent the mean ± SEM. Asterisks indicate values that are significantly different compared with the control values (*p *< 0.05)

It is well known that intracellular cAMP directly activates HCN channels. Thus, we examined intracellular adenylate cyclase (AC) activity using an AC inhibitor and a cAMP‐specific phosphodiesterase inhibitor in ANO1‐positive ICC. Basal AC activity of colonic ICC in the control group was higher than that in the small intestinal ICC. Furthermore, treatment with rolipram (10 μM), a cAMP‐specific phosphodiesterase inhibitor, showed increased AC activity, whereas SQ22536 (10 μM), an AC inhibitor, decreased AC activity in colonic ICC (Figure 4A, B, *n* = 4–7). However, rolipram or SQ22536 did not show any influence on AC activity in small intestinal ICC. For support those experiments, we tested various concentration of rolipram or SQ22536 in colonic or small intestinal ICC and found only high concentration of rolipram increased AC activity and high SQ22536 decreased AC activity in colonic ICC. However, they showed a concentration‐dependent effect on colonic ICCs (Figure 4C, D, *n*=5–6).

### 3.4 **Sulprostone increased the maximum rate of rise of resting membrane in pacemaker potentials and AC activity in colonic** ICC

**FIGURE 5:**
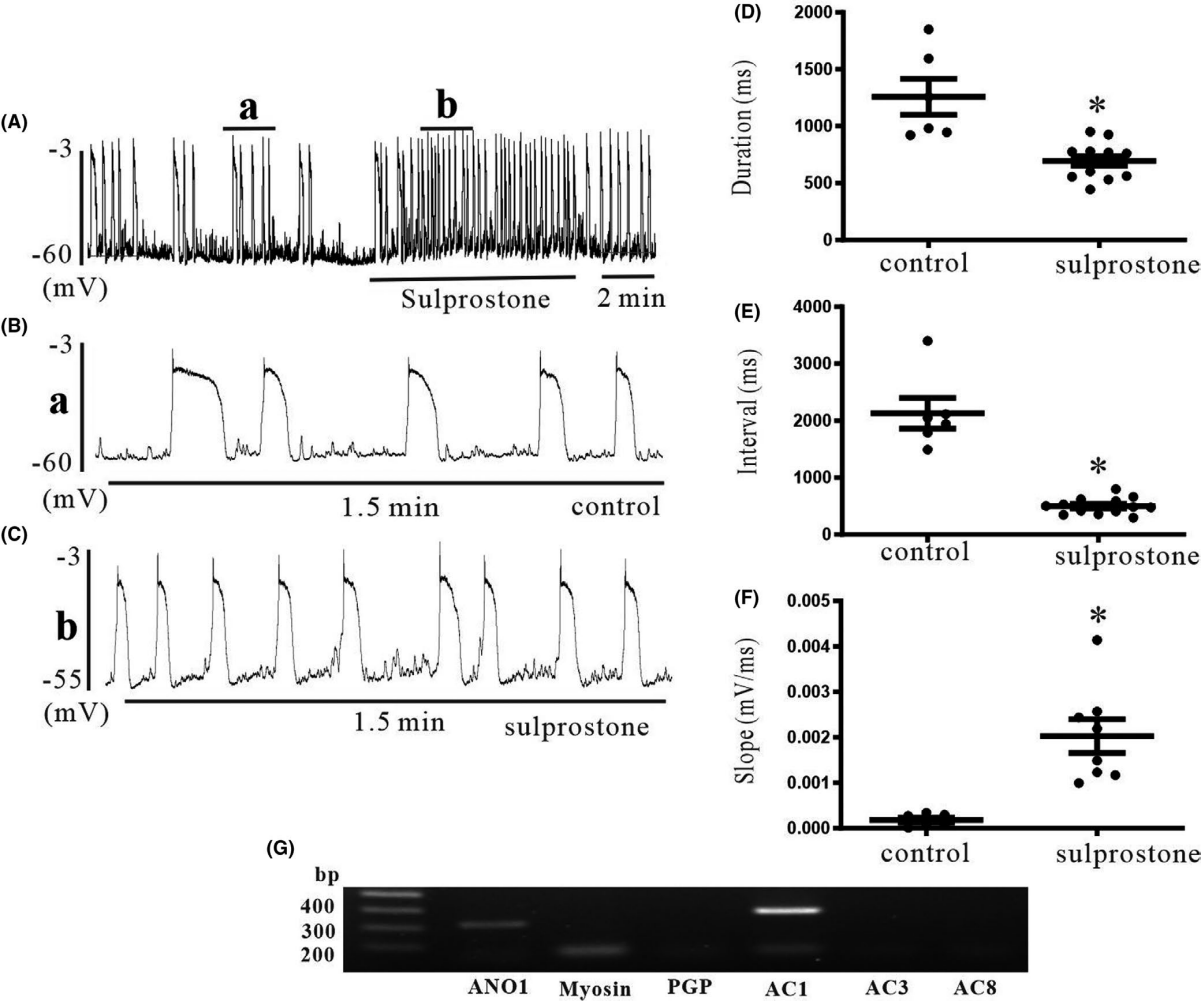
Effects of sulprostone on pacemaking potentials in colonic ICC and RT‐PCR with AC subtype‐specific primers. (A) Sulprostone increased the frequency of pacemaking potential. Small a and b were enlarged (B) and (C). (B and C) The duration and interval of pacemaking potentials were decreased by sulprostone compared with the control; however, the slope of pacemaking potential was increased by sulprostone. (D–F) Summarized data of effects of sulprostone on duration, interval and slope of pacemaking potentials in colonic ICC. The data represent the mean ± SEM. Asterisks indicate values that are significantly different compared with the control values (*p *< 0.05). (G) RT‐PCR detection and expression of AC subtype proteins in cultured ICC of mouse colon. Only AC1 subtypes were amplified from mouse colon. CON: control

Sulprostone, a prostaglandin E2‐EP3 receptor agonist, increased pacemaking potential frequency, which was blocked by HCN channel blockers in cultured colonic ICC, indicating that sulprostone activates HCN channels.^21^ In cardiac sinoatrial cells, HCN channels were involved in diastolic depolarization of action potential. Thus, to determine whether diastolic depolarization of pacemaker potential in colonic ICC was affected by sulprostone, we evaluated the maximum increase in diastolic depolarization in cultured colonic ICC. Sulprostone (100 nM, *n* = 6) increased the pacemaker potential frequency (Figure 5A). The duration and interval were decreased by sulprostone compared with the control; however, the slope of pacemaker potential was increased by sulprostone (Figure 5B, C). The summarized data on frequency increasing and duration or interval decreasing of pacemaker potential by sulprostone are shown in Figure 5D–F. In addition, to determine whether sulprostone effects were involved in intracellular cAMP formation, we measured AC activity using sulprostone. Treatment with sulprostone (100 nM, *n* = 5) showed an increase in AC activity in colonic ICC, but only in small intestinal ICC (Figure 4A, B). Additionally, Ca^2+^‐dependent AC type 1 was detected in RT‐PCR analysis (Figure 5G). These results suggest that sulprostone increases intracellular cAMP levels and leads to increased HCN channel activity in colonic ICC.

### 3.5 **5 cAMP increases intracellular Ca^2+^[Ca^2+^]_i_ oscillations in colonic** ICC

**FIGURE 6:**
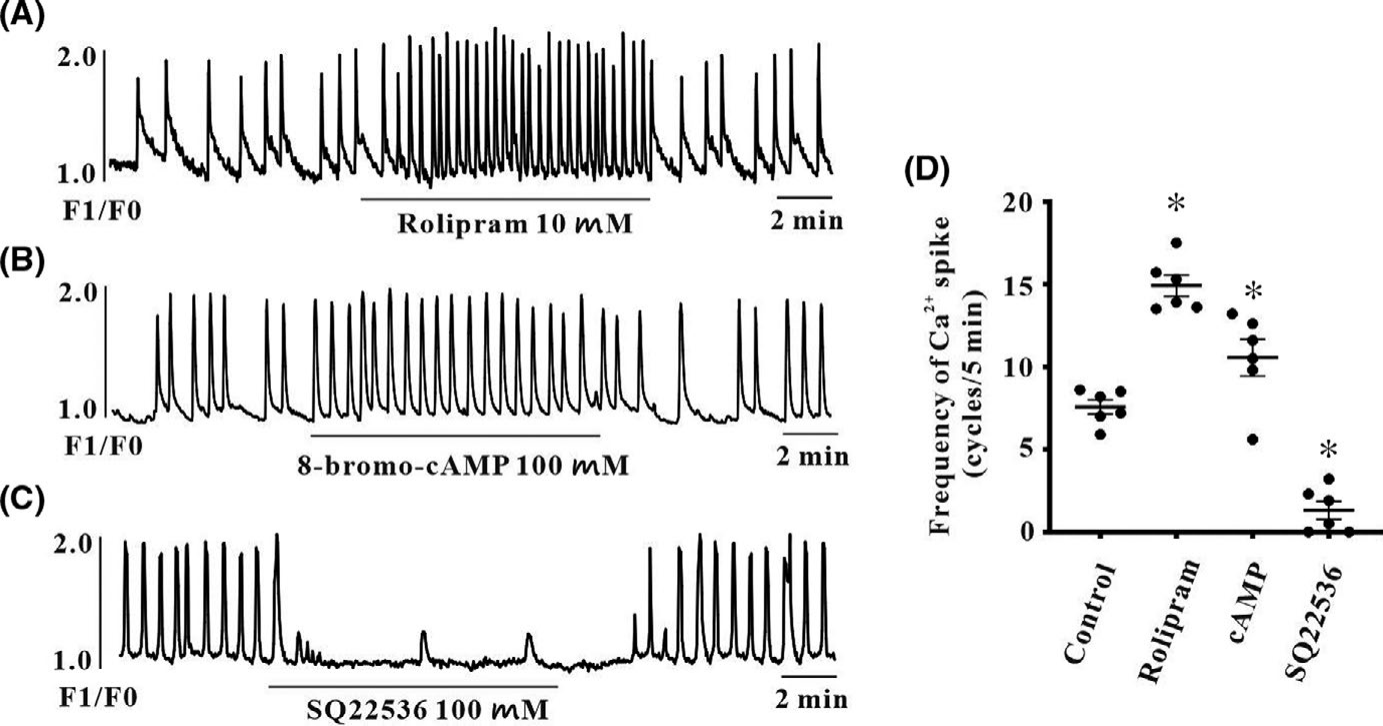
Effects of phosphodiesterase inhibitor, cAMP or AC inhibitor on intracellular Ca^2+^ ([Ca^2+^]_i_) oscillations in colonic ICC. (A and B) Both rolipram (10 μM), a phosphodiesterase inhibitor and cell‐permeable 8‐bromo‐cAMP (100 μM) increased the frequency of spontaneous [Ca^2+^]_i_ oscillations. A series of spontaneous [Ca^2+^]_i_ oscillations were observed over a time period in ICC loaded with fluo4‐AM. (C) SQ22536 (100 μM), an AC inhibitor, decreased the frequency of spontaneous [Ca^2+^]_i_ oscillations. (D) Summarized frequency changing of [Ca^2+^]_i_ oscillations by rolipram, 8‐bromo‐cAMP or SQ22536; bars represent mean ± SEM values (*n* = 6 per group). *p < 0.05 significantly different from control. cAMP: 8‐bromo‐cAMP

The activation of pacemaker channels is closely coupled with spontaneous [Ca^2+^]_i_ oscillations in ICC, and intracellular cAMP directly activates HCN channels. Thus, to evaluate whether intracellular cAMP is coupled with spontaneous [Ca^2+^]_i_ oscillations or not, we measured spontaneous [Ca^2+^]_i_ oscillations in cultured colonic ICC that are connected with cell clusters and treated with rolipram, cell‐permeable cAMP or SQ22536. Spontaneous [Ca^2+^]_i_ oscillations were observed over time. Both rolipram (10 μM, *n* = 6) (Figure 6A) and 8‐bromo‐cAMP (100 μM, *n* = 6) (Figure 6B) exhibited increased spontaneous [Ca^2+^]_i_ oscillations. But SQ22536 (100 μM, *n* = 6) (Figure 6C) decreased spontaneous [Ca^2+^]_i_ oscillations. The effects of rolipram, 8‐bromo‐cAMP or SQ22536 on [Ca^2+^]_i_ are summarized in Figure 6D.

## 4 DISCUSSION

In the present study, we confirmed the presence of HCN channels that act as pacemaker channels and contribute to the generation of spontaneous pacemaking activity in colonic ICC. HCN channels are comprised of four isoforms (HCN1‐HCN4) that show different patterns of localization in the CNS and heart.^23^, ^24^ Therefore, it seems that HCN isoforms play different physiological roles. In the GI tract, HCN channels are distributed in enteric neurons and express four channels in the myenteric plexus of the mouse colon.^25^ Yang et al.^26^ Reported that HCN2 channels are distributed only in myenteric neurons of the mouse GI tract but not necessarily in ICC. HCN2‐positive cells were labelled as cholinergic neurons via immunohistochemical studies. Thus, the authors suggest that HCN2 channels may be related to the acetylcholine release of cholinergic neurons.^26^

Recently, HCN2, HCN3 and HCN4 channels were reported in enteric neurons and ICC of normal human colons.^27^ They also found that the expression of HCN3 channels is markedly reduced not only in the aganglionic bowel but also in the ganglionic bowel of patients with Hirschsprung's disease (HSCR). In addition, they found decreased expression of ICC both in the aganglionic bowel and ganglionic bowel in patients.^28^ These findings, therefore, suggest that decreased expression of HCN3 channels and ICC may contribute to motility dysfunction in HSCR. We also reported that RT‐PCR using mouse HCN primers revealed mRNA for HCN1 and HCN3 channels in cultured ANO1‐positive ICC from mouse colon, suggesting that these channels may participate in generating pacemaker activity.^19^ However, our previous experiments were mainly pharmacological in nature. Thus, to confirm the presence of HCN channels and the functional roles of pacemaking activity, we performed immunohistochemistry studies with intact colonic tissues and functional siRNA deletion with specific nucleotides in this study.

Immunofluorescence double‐staining revealed that immunoreactivity of HCN1 and HCN3 channels was detected in ANO1‐positive colonic ICC. HCN1 channels were detected within both the muscular and myenteric layers, but HCN3 channels were detected only in the myenteric layers. HCN2 channels were expressed at the lower parts of the longitudinal layer. Thus, we performed RT‐PCR to determine whether the immunocytochemistry results were correlated. RT‐PCR analysis revealed the presence of mRNA transcripts for HCN1 and HCN3 subunits but not for HCN2 and HCN4 subunits in ANO1‐positive colonic ICC. These results were consistent with data from the immunohistochemistry studies and RT‐PCR, indicating that strong HCN channels exist in colonic ICC. The absence of HCN2 subunits in RT‐PCR indicates that HCN1 and HCN3 channels in myenteric ICC are related to the generation of pacemaker activity.

We also identified the functional roles of HCN deletion in generating pacemaker potentials in colonic ICC. HCN channels are activated at rest, and an inward Na^+^ influx leads to depolarized resting membrane potentials. Silencing of HCN1 or HCN3 channels in colonic ICC by siRNA resulted in a significant decrease in pacemaking potential frequency and hyperpolarization in the resting membrane potential compared with the control. However, the pacemaking potential frequency and resting membrane potential did not change in small intestinal ICC. Therefore, we hypothesized that the difference in pacemaking potential frequency and configuration between small intestinal and colonic ICC may be due to the basal activity of HCN channels. HCN channels are gated by intracellular cAMP directly^20^; intracellular cAMP levels are maintained by the production of adenylate cyclase (AC) and by breakdown of phosphodiesterase.^29^ Thus, to confirm that a change in basal intracellular cAMP level is coupled by HCN channel activation, we measured intracellular AC activity in colonic ICC. Our previous data showed that rolipram, a cAMP‐specific phosphodiesterase, increased pacemaking potential frequency, whereas SQ22536, an AC inhibitor, decreased pacemaking potential frequency in colonic ICCs but not in small intestinal ICC. The present study showed increased AC activity due to rolipram and decreased AC activity due to SQ22536 in colonic ICC. As the generation of pacemaker potentials was blocked by HCN channel blockers in colonic ICC, our data suggest that periodic activation of pacemaker potentials is linked to intracellular cAMP concentration oscillations.

Periodic pacemaker channel activation is closely coupled with inositol 1,3,5‐triphosphate spontaneous intracellular Ca^2+^ ([Ca^2+^]_i_) oscillations in ICC. We previously found that HCN channel blockers suppress spontaneous [Ca^2+^]_i_ oscillations in colonic ICC. In the present study, rolipram and 8‐bromo‐cAMP increased spontaneous [Ca^2+^]_i_ oscillations, indicating that HCN channel activation is closely related to intracellular cAMP levels. This suggests that basal continuous cAMP production determines the resting frequency of pacemaker potentials by binding HCN channels in colonic ICC. Both cAMP and [Ca^2+^]_i_ are ubiquitous secondary messengers that control cellular functions. There are nine types of AC (type 1 to type 9), and types 1, 3, and 8 are activated by [Ca^2+^]_i_.^30^ Therefore, we also performed RT‐PCR to determine whether Ca^2+^‐dependent AC types are involved in generating pacemaker potentials. In the present study, the mRNA transcript of AC type 1 was detected in colonic ICC, indicating that increased intracellular Ca^2+^ following activation of Ca^2+^‐dependent AC leads to the generation of pacemaker potentials in colonic ICC.

In the generation of pacemaker potentials in the sinoatrial node, HCN channels are activated at resting membrane potentials, decreasing maximal diastolic potential; therefore, they are involved in periodic diastolic depolarization.^31^ Maximal diastolic potential is increased and the slope of diastolic depolarization is decreased in HCN1 knockout mice, which thereby reduces the heartbeat. The slope of diastolic depolarization in the sinoatrial node was increased by β‐adrenoceptor activation and decreased by muscarinic activation.^32^ These observations suggest that neurotransmitters and endogenous substances can influence firing rates by increasing or decreasing the slope of diastolic depolarization via HCN channels. We recently reported that sulprostone, a PGE_2_‐EP_3_ agonist, enhanced pacemaking potential frequency and spontaneous [Ca^2+^]_i_ oscillations and these effects were blocked by HCN channel blockers. However, sulprostone had no effects on pacemaker potentials in small intestinal ICC.^21^ Thus, in this study, to confirm the functional data of sulprostone, we measured the maximum rate of rise of the resting membrane of pacemaker potentials of colonic ICC. Sulprostone increased the maximum rate of rise of the resting membrane of pacemaker potentials and decreased the duration and interval of pacemaker potentials. In addition, sulprostone increased AC activity in colonic ICC but not in small intestinal ICC. These results suggest that periodic activation of HCN channels may be involved in diastolic depolarization like cardiac HCN channels, and sulprostone activates HCN channels through an increase in intracellular cAMP levels via AC activation in colonic ICC. It has been reported that sulprostone increases bladder excitability by activating HCN channels.^33^

Pacemaker channel activation‐induced inward currents lead to depolarization of the membrane, which is followed by activation of T‐type Ca^2+^ channels^34^, ^35^ and initiating rhythmic activity in ICC. Until now, ANO1 and T‐type channels have been considered representative ion channels associated with the generation of pacemaker activity. However, there is no argument that the initiation of pacemaker activity involves the release of [Ca^2+^]_i_. Our results support the hypothesis that HCN channels can regulate pacemaker activity via [Ca^2+^]_i_. Therefore, our study suggests that HCN channel has the role for generation of pacemaker activity and this indicates HCN can be a candidate as pacemaker channel in colonic ICC, together with ANO1. Additionally, tonic activation of HCN channels at resting membrane potential induces inward currents and leads to depolarization of the membrane. This is followed by the activation of T‐type Ca^2+^ channels, initiating pacemaker potentials in colonic ICC.

In conclusion, the results presented here confirm our previous hypothesis that HCN channels are present in colonic ICC and are involved in generation of spontaneous pacemaker activity. Therefore, therapeutic targeting of HCN channels may be effective in decreasing colonic motility disorders.

## CONFLICT OF INTEREST

The authors confirm that this article and its authors have no conflicts of interest to declare.

## AUTHOR CONTRIBUTIONS

**Seok Choi:** Conceptualization (equal); Formal analysis (equal); Writing – original draft (equal). **Hyunhyo Seo:** Conceptualization (equal); Data curation (equal). **Kyungmin Lee:** Investigation (equal); Methodology (equal). **Dong Hoon Shin:** Investigation (equal); Methodology (equal). **Mei Jin Wu:** Investigation (equal); Methodology (equal). **Wenhao Wu:** Investigation (equal); Methodology (equal). **Xingyou Huang:** Investigation (equal); Methodology (equal). **Jingwei Zhang:** Investigation (equal); Methodology (equal). **Chansik Hong:** Investigation (equal); Methodology (equal). **Jae Yeoul Jun:** Conceptualization (equal); Data curation (equal); Formal analysis (equal); Funding acquisition (equal); Project administration (equal); Supervision (equal); Writing – original draft (equal); Writing – review & editing (equal).

## Supporting information

Figure S1

## Data Availability

Data openly available in a public repository that issues datasets with DOIs.
