# Supplementary figures and images for: Hyperpolarization‐activated cyclic nucleotide‐gated channels working as pacemaker channels in colonic interstitial cells of Cajal
Source: J Cell Mol Med. 2021 Nov 29;26(2):364–74. doi: 10.1111/jcmm.17087 (PMC8743669; doi:10.1111/jcmm.17087)

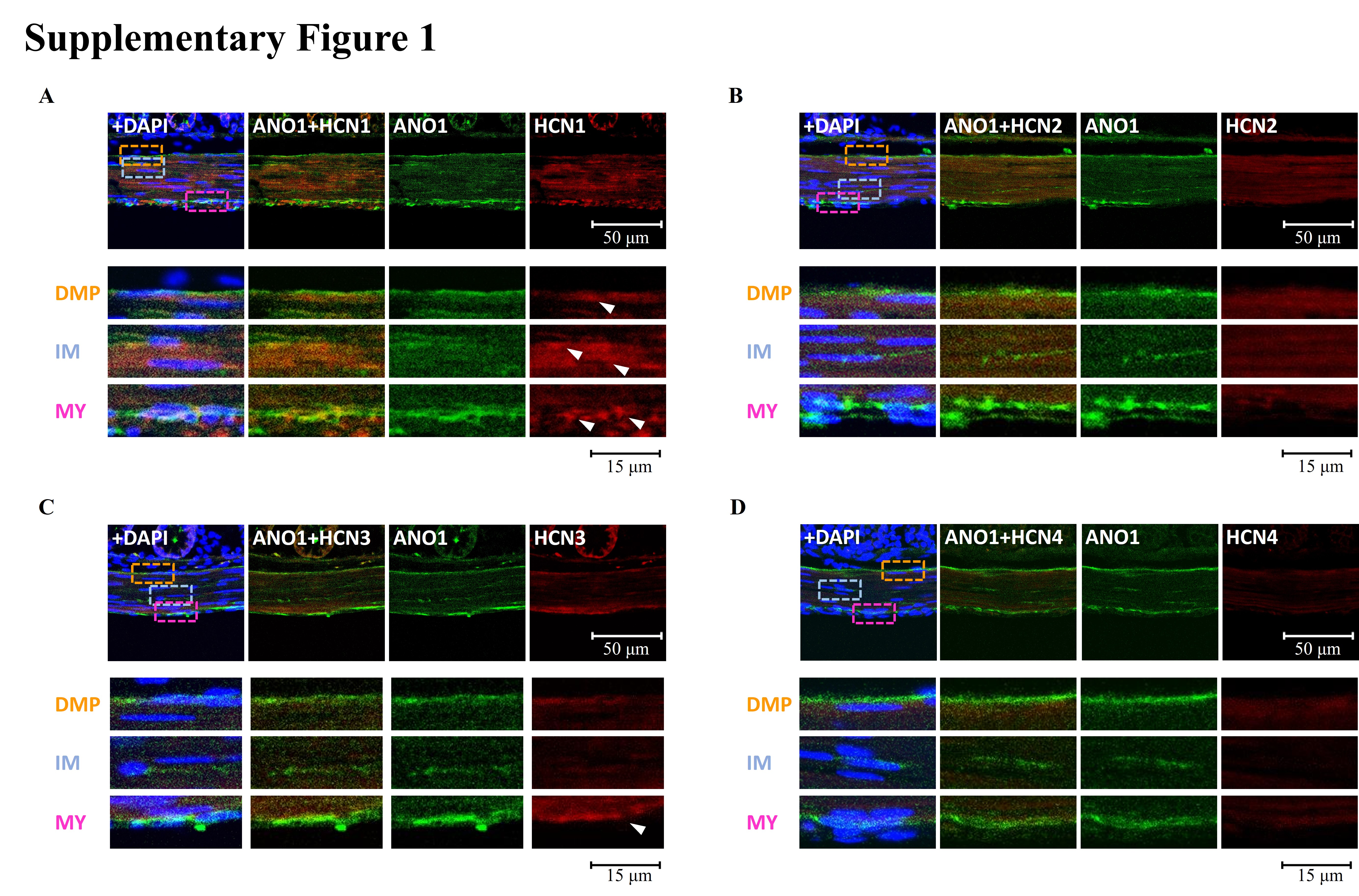

Supplement: Supplementary file 1 — Figure S1 [file JCMM-26-364-s001.jpg]
